# Changing Features of COVID-19: Characteristics of Infections with the SARS-CoV-2 Delta (B.1.617.2) and Alpha (B.1.1.7) Variants in Southern Italy

**DOI:** 10.3390/vaccines9111354

**Published:** 2021-11-18

**Authors:** Daniela Loconsole, Francesca Centrone, Caterina Morcavallo, Silvia Campanella, Marisa Accogli, Anna Sallustio, Davide Peccarisi, Angela Stufano, Piero Lovreglio, Maria Chironna

**Affiliations:** 1 Department of Biomedical Sciences and Human Oncology-Hygiene Section, School of Medicine, University of Bari, 70124 Bari, Italy; daniela.loconsole@uniba.it (D.L.); francesca.centrone.fc@gmail.com (F.C.); caterinamorcavallo@gmail.com (C.M.); campanella.silvia@libero.it (S.C.); accoisa@gmail.com (M.A.); davidepec@libero.it (D.P.); 2 Hygiene Unit, Azienda Ospedaliero Universitaria Consorziale Policlinico di Bari, 70124 Bari, Italy; annasallustio@libero.it; 3 Interdisciplinary Department of Medicine-Section of Occupational Medicine, School of Medicine, University of Bari, 70124 Bari, Italy; angela.stufano@uniba.it (A.S.); piero.lovreglio@uniba.it (P.L.)

**Keywords:** SARS-CoV-2 Delta variant, SARS-CoV-2 Alpha variant, COVID-19 vaccine, surveillance, epidemiology, hospitalization, Italy

## Abstract

Differences in the demographic and clinical characteristics of patients infected with the Alpha and Delta SARS-CoV-2 variants of concern in a large region of Southern Italy were assessed. Two cohorts of positive patients were compared. The Alpha group consisted of 11,135 subjects diagnosed between 21 March and 21 April 2021, and the Delta group consisted of 499 positive subjects diagnosed between 21 July and 21 August 2021. A descriptive and statistical analysis of the demographic and clinical characteristics of the two groups was performed. The proportion of patients with mild and moderate infections was significantly higher in the Delta than in the Alpha group (*p* < 0.001). In fully vaccinated patients, the proportion of symptomatic individuals was significantly higher in the Delta than in the Alpha group. The Delta group showed odds ratios of 3.08 (95% CI, 2.55–3.72) for symptomatic infection and 2.66 (95% CI, 1.76–3.94) for hospitalization. Improving COVID-19 vaccination rates is a priority, since infection with the SARS-CoV-2 Delta variant has a significant impact on patient outcomes. Additional targeted prevention strategies such as social distancing, the use of masks in indoor settings irrespective of vaccination status, and the use of a sanitary passport could be crucial to contain further spread of SARS-CoV-2 infection.

## 1. Introduction

The widespread circulation of SARS-CoV-2 since the beginning of the pandemic has allowed the virus to constantly mutate, resulting in the emergence of new variants. The genetic changes that provide the virus with a selective advantage have led to the spread of several variants of concern (VOC). These variants are characterized by increased transmissibility, virulence, and/or pathogenicity [1]. VOCs are also linked to decreases in the effectiveness of public health measures, including diagnostics, vaccines, and therapeutics, and to rapid epidemiological changes [1]. Constant monitoring and rapid assessment of genetic changes are required to detect the spread of these variants [2]. Improvements in local genomic surveillance and increased levels of concern about the introduction of new variants, the amount of international travel among affected countries, and local transmission, have increased awareness of the spread of VOCs [3]. 

The first identified SARS-CoV-2 VOC was the Alpha variant (Pango lineage B.1.1.7), which was first detected in the United Kingdom in September 2020 [4] and became the predominant lineage by early February 2021, accounting for 95% of cases [5]. This variant was first identified in Italy in December 2020 [6,7,8], becoming the dominant strain within a few months. This was accompanied by a rapid increase in the number of symptomatic cases and hospitalizations, resulting in significant pressure on the healthcare system [9,10,11]. Other VOCs detected after the identification of the B.1.1.7 variant included the Beta variant (Pango lineage B.1.351), first detected in South Africa in October 2020, and the Gamma variant (Pango lineage P.1), initially identified in Brazil [1]. The Delta variant (Pango lineage B.1.617.2), first identified in India in December 2020 [12], was designated a VOC in May 2021 and was associated with epidemic rebounds in several countries [1]. Currently, the Delta variant is the predominant strain identified worldwide [13]. When compared with other variants, this VOC is characterized by a transmission advantage, an increased risk of hospitalization [14], a higher virulence [15,16] found mostly in unvaccinated and not-fully vaccinated people, and a lower vaccine effectiveness against symptomatic COVID-19 [17]. In particular, the B.1.617.2 variant has been estimated to be twofold more transmissible than previous circulating variants [14] due to the presence of L452R and P681R mutations in the receptor binding domain of the spike protein [18]. However, the rates of hospitalizations and deaths in countries with high vaccination coverage that are experiencing Delta variant waves of infection have been lower than those experienced with the Alpha variant [19].

In Italy, the Delta VOC started circulating in May 2021, showing a prevalence of 1% in a national flash survey [20]. However, within a few months the B.1.617.2 lineage had almost completely replaced the B.1.1.7 lineage, with the prevalence of SARS-CoV-2 B.1.617.2 being 99.7% at the end of August 2021 [21]. After the spread of the Delta variant, the Alpha variant is no longer considered a VOC [22].

Currently, little is known about the impact of the Delta VOC on the national health system, especially in countries with early and extensive vaccination campaigns. The present study was designed to determine differences in the demographic and clinical characteristics of patients infected with the SARS-CoV-2 Alpha and Delta variants, especially the risk of hospitalizations and deaths, in the large Apulia region of Southern Italy.

## 2. Materials and Methods

### 2.1. Study Population

The COVID-19 national surveillance system in Italy includes integrated epidemiological and microbiological surveillance [23]. All epidemiological and molecular data of patients with a confirmed diagnosis of SARS-CoV-2 infection are uploaded to the national surveillance platform. 

The present study compared two patient cohorts. The first cohort consisted of 11,135 subjects who were diagnosed with SARS-CoV-2 infection between 21 March and 21 April 2021, when the estimated prevalence of the Alpha variant was 91.6% in Italy and 98.3% in the Apulia region [24]. The second cohort consisted of 499 SARS-CoV-2 positive patients initially diagnosed between 21 July and 21 August 2021, when the estimated prevalence of the Delta variant was 99.7% in Italy and 100% in the Apulia region [21]. All of the enrolled patients in both cohorts had been diagnosed at the Laboratory of Molecular Epidemiology and Public Health of the Hygiene Unit of the Policlinico Hospital of Bari, the Regional Reference Laboratory for COVID-19. This laboratory processes nasopharyngeal swabs collected from both hospitalized and non-hospitalized patients, covering 60% of the population of the province of Bari (1,230,205 inhabitants). Nasopharyngeal swabs (UTM, FLOQ Swabs TM, Copan Italia, Brescia, Italy) were subjected to molecular testing using a commercial, real-time PCR assay, as previously described [9].

The demographic characteristics of all patients, as well as information about symptoms, vaccinations, and hospitalizations for COVID-19, were recorded. Cases were defined according to the National Institutes of Health (NIH) clinical staging of COVID-19 disease [25]. In particular, patients infected with SARS-CoV-2 were classified as “asymptomatic” if no signs or symptoms of COVID-19 were present; “mild” if symptoms such as fever, cough, sore throat, malaise, headache, muscle pain, nausea, vomiting, diarrhea, or loss of taste and smell were present, but there was no evidence of shortness of breath, dyspnea, or abnormal chest imaging; “moderate” if there was evidence of lower respiratory disease during clinical assessment but hospitalization was not required; “severe” if SpO2 was <94%, respiratory rate was >30 breaths/min, or chest imaging showed >50% lung infiltrates, with signs and symptoms of respiratory disease severe enough to require hospitalization; and “critical” if respiratory failure, septic shock, and/or multiple organ dysfunction had occurred, with the patient requiring admission to the intensive care unit [25]. In the present study, patients classified as severe and critical cases were combined into a single group labeled “severe”.

### 2.2. Data Analysis

Patients were classified by the worst clinical condition they experienced, as registered in the national COVID-19 surveillance platform. Patients were categorized as unvaccinated (≤14 days since the first dose of any COVID-19 vaccine), partially vaccinated (>14 days after the first dose of any COVID-19 two-dose vaccine or ≤14 days after completing the vaccination schedule for a single-shot vaccine or a two-dose vaccine), and fully vaccinated (>14 days after a single-shot vaccine, or after the second shot of a two-dose vaccine). Because of its small sample size, the group of partially-vaccinated patients was not included in this analysis. Data were reported as proportions and 95% confidence intervals (CI), and compared by the Chi-squared or Fisher exact test. A multivariate logistic regression model was used to explore associations among clinical status, hospitalizations, and infections with the Alpha or Delta variant. Data were analyzed using STATA 12.1 software, with a *p* < 0.05 considered statistically significant.

### 2.3. Ethics Statement

Approval by the Ethics Committee was waived because the activities described in this study were part of the legislated mandate of the Health Promotion and Public Health Department of the Apulia region (Italy). All procedures were performed in accordance with the Declaration of Helsinki, as revised in 2013, for research involving human subjects.

## 3. Results

Overall, 11,634 SARS-CoV-2 positive subjects residing in the province of Bari were enrolled. For the purpose of the study, the first group (*n* = 11,135) was labeled “Alpha”, whereas the second group (*n* = 499) was labeled “Delta”. The main reason for swab collection from patients in the Alpha group was contact tracing (45.1%), followed by assessment of symptoms (26.7%), and screening (26.4%). The main reason for swab collection from patients in the Delta group was also contact tracing activity (50.1%), followed by screening (28.3%), and assessment of symptoms (17.2%). The reasons for swab collection were not known for 1.8% of the patients in the Alpha group and 4.4% of the patients in the Delta group. Of the patients in the Alpha group, 96.1% (*n* = 10,702) were unvaccinated, 3.2% (*n* = 354) were partially vaccinated, and 0.7% (*n* = 79) were fully vaccinated, whereas, of the patients in the Delta group, 61.7% (*n* = 308) were unvaccinated, 10.8% (*n* = 54) were partially vaccinated, and 27.5% (*n* = 137) were fully vaccinated. The demographic and clinical characteristics of the two groups are reported in Table 1. 

The proportion of subjects infected with the SARS-CoV-2 Alpha variant was significantly higher in patients aged ≥36 years, whereas the proportion of subjects infected with the Delta variant was significantly higher in patients aged <36 years. Moreover, the proportion of patients with mild and moderate infections was significantly higher in the Delta than in the Alpha group (*p* < 0.001). In addition, the proportion of patients with severe COVID-19 was higher in the Delta than in the Alpha group, although the difference was not statistically significant. Patients infected with the Delta variant were more likely to be hospitalized (*p* = 0.015), whereas death rates in the two groups did not differ significantly. 

Table 2 shows a comparison of the clinical status of the two groups according to age and vaccination status. A comparison of unvaccinated patients showed that the proportion of symptomatic individuals was significantly higher in the Delta than in the Alpha group, except for patients aged >65 years. In fully vaccinated patients, the proportion of symptomatic individuals was significantly higher in the Delta than in the Alpha group, for all the analyzed age groups. Multivariate logistic regression analysis was performed to evaluate the clinical status and risk of hospitalization in the two groups, adjusted for age, sex, and vaccination status. For the purpose of the analysis, comorbidities were not considered since this data was not available for most of the enrolled patients. Specifically, the Delta group showed odds ratios (OR) of 3.08 (95% CI, 2.55–3.72) for symptomatic infection and 2.66 (95% CI, 1.76–3.94) for hospitalization.

## 4. Discussion

Since its first appearance in India, the Delta variant has spread rapidly worldwide, being responsible for a surge in infection cases [26]. The impact of the Delta variant in India has been considerable because of its high infection rate and overwhelming burden on hospitals, resulting in shortages of supplies and life-saving equipment [27]. Therefore, concerns have arisen regarding the ability of this variant to evade SARS-CoV-2 vaccine-induced immunity [28]. 

The present study was designed to evaluate the impact of the Delta variant in a large region of Southern Italy by assessing rates of symptomatic disease, hospitalizations, and deaths. The high percentage of subjects aged ≥36 years infected with the Alpha variant may have been due to the higher vaccine coverage in adults and older age groups during the period of widespread circulation of the Delta variant [29]. The B.1.617.2 SARS-CoV-2 variant has been reported as responsible for the increases in the numbers of COVID-19 cases in children and adolescents [30]. In particular, the incidence of COVID-19 cases among subjects aged 0–17 years the United States was 10-fold higher in August 2021 than in June 2021 [30].

Considering the whole cohorts, our results showed a higher risk of severe disease in Delta variant cases compared to Alpha variant cases, although the difference was not statistically significant. This data could be explained by the higher percentage of older patients in the Alpha group, who are more likely to get severely ill from COVID-19 [31], the low rate of vaccination coverage [32], and the over-represented testing activity in symptomatic patients during the period characterized by the spread of the Alpha variant. Moreover, our evaluation of fully vaccinated patients showed that the proportion of symptomatic subjects was higher in the Delta than in the Alpha group. This may be associated with the reduction in neutralizing activity against the Delta VOC in fully vaccinated subjects [26,33]. In particular, neutralizing activity was twofold lower in fully vaccinated than in unvaccinated patients who were infected with the Delta variant, but 1.7-fold lower in fully vaccinated than in unvaccinated patients infected with the Alpha variant [26]. Nevertheless, it is not clear whether the reduction in neutralizing activity can alter the effectiveness of COVID-19 vaccines. The effectiveness of vaccine against infection with the Delta variant has been reported to be significantly lower than that against infection with the Alpha variant, both in fully vaccinated and partially vaccinated subjects [17]. By contrast, other studies have reported only a modest decrease in vaccine effectiveness against infection with the Delta variant [34]. Further studies are needed to clarify this crucial issue.

The emergence of the Delta variant might not be the main driver of the reported decline in effectiveness of the COVID-19 vaccines [35,36]. Although the BNT162b2 mRNA COVID-19 vaccine was reported to be highly effective for the first six months after subjects were fully vaccinated, even during the period of predominant circulation of the Delta variant, the subsequent reduction in vaccine effectiveness was likely due to waning immunity, rather than an escape from vaccine protection [35]. Based on the experience of other countries with widespread dissemination of the Delta variant that is triggering another epidemic wave despite high vaccination coverage [37], the European Medicine Agency (EMA) has recommended booster doses to restore the high levels of protection that were observed early in the vaccination program [38]. Booster doses of COVID-19 vaccine have also been strongly recommended in Italy [39].

In our study, multivariate analysis showed that patients infected with the Delta variant were at more than threefold greater risk of symptomatic disease and more than twofold greater risk of hospitalization than patients infected with the Alpha variant. These findings regarding hospitalization agree with the results of a previous study, which found that patients infected with the B.1.617.2 variant were at threefold greater risk of hospitalization than patients infected with the Alpha variant [2]. The latter study by Twohig et al. was conducted in England between March 29 and May 23, 2021, and compared two cohorts of patients infected with Alpha and Delta variants in the same period of time [2]. Some reduction in vaccine effectiveness against hospital admissions has been observed in people >65 years and fully vaccinated with the BNT162b2 COVID-19 vaccine six months after the administration of the second dose [35].

Similarly, vaccination reduces the risks of hospital admission in patients infected with the Delta and Alpha variants [40]. Our study did not compare rates of hospitalization by variant and vaccination status because the number of vaccinated patients hospitalized with the Alpha variant was too low to determine whether the risk of hospital admission was different among the two groups. This was likely due to the makeup of the Alpha group, which consisted of COVID-19 patients infected from 21 March to 21 April 2021, a period characterized by widespread circulation of the Alpha variant, but a low rate of vaccination [32].

Although this study showed that infection with the SARS-CoV-2 Delta variant had a significant impact on patient outcomes, several factors may affect the accuracy of these findings. First, SARS-CoV-2 positive samples were not subjected to whole-genome sequencing (WGS), as the two cohorts were selected based on the prevalence of the two variants, not by sample sequencing. In fact, the study compares two different periods of time. However, the prevalence of each variant during the two periods was nearly 100%. In addition, sequencing data available on the GISAID database shows that, during the study periods, very few sequenced samples were VOCs other than Alpha or Delta. Second, the sample sizes of the two cohorts differed markedly. Nevertheless, these findings reflect the rates of SARS-CoV-2 infection during the two time periods. The major strength of our study is to gain insight into some important epidemiological aspects of Delta variant spread in Italy. Our findings, along with the current epidemiology of SARS-CoV-2 infection, could represent the base for public health decisions in our country. The high transmissibility of the Delta variant, the lower vaccine effectiveness, and the current increases in contact rates are likely to lead to an increased transmission of the infection [41]. Moreover, according to some mathematical models, the circulation of the Delta variant might not fully allow the removal of non-pharmaceutical interventions (NPIs) without a new wave of hospital admissions and deaths, despite high vaccination coverage [41]. 

Improving COVID-19 vaccination rates, including in hard-to-reach communities, is a priority, especially because the Delta variant is associated with higher risks of symptomatic COVID-19 and hospitalization. Vaccination is the key strategy (even with extra and booster doses) for preventing infection, severe symptoms, hospitalizations, and deaths associated with SARS-CoV-2 infections, including in children not yet eligible for vaccination [30]. However, due to the high transmissibility of the Delta variant, additional targeted prevention strategies such as social distancing, the use of masks in indoor settings irrespective of vaccination status, and the use of a sanitary passport (vaccination or periodic SARS-CoV-2 testing) could be crucial to contain the spread of infection during the autumn and winter respiratory virus seasons.

## Figures and Tables

**Table 1:** Demographic and clinical characteristics of patients who were positive for the Alpha and Delta variants.

	Alpha		Delta		*p*-Value
	*n*	%	*n*	%	
Total patients enrolled	11,135		499		
Sex					
Female	5863	52.7%	237	47.5%	0.024
Male	5272	47.3%	262	52.5%	
Age groups (years)					
0–4	254	2.3%	22	4.4%	0.002
5–16	1376	12.4%	82	16.4%	0.007
17–35	2574	23.1%	175	35.1%	<0.001
36–65	4965	44.6%	172	34.5%	<0.001
>65	1966	17.7%	48	9.6%	<0.001
Clinical status					
Asymptomatic	7155	64.3%	208	41.7%	<0.001
Mild	2369	21.3%	188	37.7%	<0.001
Moderate	1183	10.6%	79	15.8%	<0.001
Severe	428	3.8%	24	4.8%	0.274
Hospital admission	464	4.2%	32	6.4%	0.015
Deaths	197	1.8%	7	1.4%	0.542

**Table 2:** Comparison of the clinical status in patients who were positive for the Alpha and Delta variants by age group and vaccination status.

		Alpha		Delta		*p*-Value
		*n*	%	*n*	%	
Unvaccinated	0–4 year					
	Asymptomatic	178	70.1%	10	45.5%	0.017
	Symptomatic	76	29.9%	12	54.5%	
	5–16 year					
	Asymptomatic	1045	75.9%	33	42.9%	<0.001
	Symptomatic	331	24.1%	44	57.1%	
	17–35 year					
	Asymptomatic	1784	70.8%	47	39.2%	<0.001
	Symptomatic	737	29.2%	73	60.8%	
	36–65 year					
	Asymptomatic	2975	62.5%	30	39.5%	<0.001
	Symptomatic	1787	37.5%	46	60.5%	
	>65 year					
	Asymptomatic	880	49.2%	4	30.8%	0.266
	Symptomatic	909	50.8%	9	69.2%	
Fully vaccinated	17–35 year					
	Asymptomatic	11	73.3%	9	26.5%	0.004
	Symptomatic	4	26.7%	25	73.5%	
	36–65 year					
	Asymptomatic	35	76.1%	34	47.9%	0.002
	Symptomatic	11	23.9%	37	52.1%	
	>65 year					
	Asymptomatic	16	88.9%	13	40.6%	0.001
	Symptomatic	2	11.1%	19	59.4%	

## Data Availability

All data are available from the corresponding author by e-mail request.
